# Editorial: Proanthocyanidins and isoflavonoids

**DOI:** 10.3389/fpls.2024.1498989

**Published:** 2024-10-02

**Authors:** Li Tian, Deyu Xie

**Affiliations:** ^1^ Department of Plant Sciences, University of California, Davis, Davis, CA, United States; ^2^ Department of Plant and Microbial Biology, North Carolina State University, Raleigh, NC, United States

**Keywords:** proanthocyanidin, condensed tannin, isoflavonoid, flavonoid, anthocyanin

Proanthocyanidins (PAs), also known as condensed tannins (CTs), are polyphenolic compounds produced in a wide range of plants. They are recognized for their antioxidant properties, functions in plant defense, and contributions to the astringency and nutritional quality of foods. Isoflavonoids, a subclass of flavonoids primarily found in legumes, play important roles in plant defense and symbiotic interactions with nitrogen-fixing bacteria. Recent advances have greatly enhanced our understanding of the biosynthesis of PAs and isoflavonoids. For example, studies have uncovered new roles for anthocyanidin reductase (ANR), leucoanthocyanidin reductase (LAR), and anthocyanidin synthase (ANS) in PA formation, as well as novel mechanisms governing PA polymerization and regulation. Similarly, progress in understanding enzyme structures and regulatory pathways has shed light on previously unknown aspects of isoflavonoid biosynthesis.

In collaboration with the Phytochemical Society of North America (PSNA), mentees and colleagues of Professor Richard A. Dixon have compiled this Research Topic to honor his retirement and celebrate his exceptional contributions to the study of PAs and isoflavonoids. The Research Topic includes five original research articles, two mini-reviews, and four reviews, which are organized around the following themes.

## Biosynthesis, transport, and regulation of isoflavonoids

In their comprehensive review, Wang et al. highlighted major developments in understanding key enzymes, transporters, and regulatory mechanisms involved in isoflavonoid biosynthesis. They discussed strategies, technologies, and resources for enhancing isoflavonoid levels in leguminous and non-leguminous plants through metabolic engineering. Modifications such as glycosylation and methylation of core structures are important for producing unique isoflavonoids with health-promoting activities in the traditional Chinese medicinal plant *Pueraria lobata*. Li and Zhang’s review summarized recent research on these modifications and the relevant biosynthetic enzymes in *P. lobata*. In a related review, Yang and Wang focused on recent progress in isoflavonoid biosynthesis, regulation, transport, and functions in soybeans. They also suggested exploring new factors in isoflavonoid metabolism and plant-microbe interactions.

Isoflavonoids are synthesized from phenylalanine via the phenylpropanoid and downstream pathways. Clayton et al. built on earlier research on soybean AROGENATE DEHYDRATASE (*Gm*ADT) isozymes—which catalyze the final step of phenylalanine biosynthesis through the arogenate pathway—and identified additional *Gm*ADT isoforms. Their analyses of gene structure, phylogeny, expression patterns, subcellular localization, and protein interactions of these putative *Gm*ADTs suggested a role in the isoflavonoid metabolon. Interestingly, some *Gm*ADTs exhibited PREPHENATE DEHYDRATASES (PDT) activity in the prephenate pathway for phenylalanine biosynthesis in a yeast complementation assay. Future studies could explore how different *Gm*ADTs contribute to flavonoid and isoflavonoid metabolons and the extent to which tissue-specific expression determines these functions.

## Flavonoids in citrus fruits and anthocyanins in mung beans

Rootstock grafting and fruit thinning are commonly adopted practices for varietal improvement in citrus, such as for increasing functional compounds including flavonoids, phenolic acids, and terpenes. Li S. et al. used high performance liquid chromatography (HPLC) to investigate the impact of different rootstocks on functional compounds, antioxidant capacity, and fruit quality at six developmental stages of ‘Orah’ fruit. They found that certain rootstocks significantly enhance sensory quality, functional constituents, and antioxidant properties of the fruit. They also identified key developmental stages for fruit pruning, when young fruits are abundant in functional compounds suitable for extraction. This study provided useful insights for improving orchard management practices in citrus. In parallel, Li C. et al. focused their study on mung bean, a dual-use crop valued for food and medicinal uses. Through comparative transcriptome and metabolite analysis, they identified transcripts with significantly higher accumulation in an anthocyanin-rich variety, correlating with elevated anthocyanin levels. In particular, two transcription factors, *Vr*MYB3 and *Vr*MYB90, were found to potentially increase anthocyanin levels by upregulating key biosynthetic genes. These insights into anthocyanin regulation may inform breeding efforts in mung beans.

## Pigments shaping the color palette of cotton

Naturally colored cotton (NCC) varieties come in shades ranging from green to brown; their PAs contribute to both pigmentation and UV protection in fibers. These varieties also exhibit resistance to insects and diseases, as well as tolerance to salt and drought stresses. In their review, Naoumkina et al. discussed the molecular mechanisms underlying pigment production in naturally-colored brown cotton (NBC), challenges in breeding NCC with diverse colors without compromising fiber quality, and the potential of using flame-retardant NCC varieties for textile applications. To address the knowledge gap regarding the correlation between pigmentation and flame resistance in NBC, Hinchliffe et al. conducted a comparative transcript and metabolite analysis of developing fibers from brown (MC-BL) and white (MC-WL) cotton near isogenic lines differing at a single locus. Their study revealed significant differences in metabolite profiles and gene expression during key stages of fiber development. Notably, MC-BL showed higher accumulation of the phenylpropanoid pathway derivatives and organic acids from the citric acid cycle, while amino acid levels were lower compared to MC-WL. Further research is needed to understand how these differentially accumulated metabolites contribute to the flame-retardant properties of NBC fibers.

## PA biosynthesis and perspectives

PAs, anthocyanins, and flavonols largely impact the nutritional and sensory qualities of grape berries and red wine. Shi et al. reviewed how environmental factors, such as water availability, light exposure, and temperature, as well as their interactions with phytohormones, affect the composition and content of these compounds, with the goal of providing a better understanding of the underlying regulatory mechanisms. While PAs are often found in the fruit peels and seeds of plants, such as grapes, they are also present in the vegetative tissues, including the roots, of woody plants. Westley et al. demonstrated that CTs (i.e., PAs) are predominantly located in younger white roots and at the tips of hybrid poplar (*Populus tremula* x *alba*) roots. They observed higher concentrations of CTs in root cap and epidermal cells, and lower levels in older roots and those with secondary growth. CTs are most concentrated in the insoluble fraction within the cork zone, and their accumulation correlates with increased ammonium (NH_4_^+^) uptake near the root tip, while nitrate (NO_3_^-^) and calcium (Ca^2+^) uptake remains consistent. To further investigate the functional role of CTs in roots, transgenic poplar plants with modified CT profiles could be utilized for direct testing.

Lu revisited questions raised about PA biosynthesis two decades ago and provided an update on our current understanding, particularly regarding precursors, intermediates, and polymerization reactions. However, questions remain concerning the intracellular transport and deposition of PAs, as well as the subcellular localization of enzymes involved in PA biosynthesis. The mini-review also outlined future research directions, including the exploration of additional model species, other forms of PA, factors affecting PA biosynthesis, and the extent of PA polymerization.

Looking ahead, understanding the spatial distribution and intracellular dynamics of PA and isoflavonoid biosynthesis remains a priority for future research. Additionally, elucidating the interplay between environmental factors and the metabolism of PAs and isoflavonoids could offer valuable insights for improving agricultural traits and nutritional quality. Advanced biochemical, genetic, and omics tools, combined with metabolic engineering approaches, will play a key role in bridging these research gaps and translating findings into practical applications.

